# “A sweating moment”: impact of disclosure in cancer care on LGBTQI patient satisfaction

**DOI:** 10.1007/s11764-024-01677-1

**Published:** 2024-09-21

**Authors:** Rosalie Power, Jane M. Ussher, Kimberley Allison, Alexandra Hawkey, Janette Perz

**Affiliations:** Translational Health Research Institute, Western Sydney University, Sydney, Australia

**Keywords:** Cancer, LGBTQI, Satisfaction, Disclosure, Outness

## Abstract

**Purpose:**

Lesbian, gay, bisexual, trans, queer, and intersex (LGBTQI) people face unique challenges in cancer care. This mixed methods study examined LGBTQI patients’ satisfaction with cancer care and factors associated with satisfaction, including experiences of LGBTQI disclosure. The study also explored what helps to facilitate safe disclosure and improve satisfaction with care for this population.

**Methods:**

We used a mixed methods approach, involving 430 surveys and 104 semi-structured interviews with LGBTQI people with cancer (16–92 years) with various cancer types, sexuality and gender identities, ages, and people with intersex variations.

**Results:**

Most participants reported being satisfied with their cancer care (*n* = 300, 76.3%) and had disclosed their LGBTQI sexuality or gender identity or intersex variations to at least some of their cancer healthcare professionals (HCPs) (*n* = 357, 87.1%). Satisfaction with care was higher with more disclosure to HCPs, HCP acknowledgment of partner/s and support people, and the ability to find LGBTQI specific information about cancer; it was lower with reports of discrimination in cancer care. Qualitative analysis identified that some participants were always out to HCPs, but others felt burdened with the responsibility and emotion work of disclosure and feared negative responses. Same-gender intimate partners facilitated disclosure and need to be respected within cancer care.

**Conclusions:**

HCPs need to take the lead in facilitating LGBTQI disclosure in cancer care. Targeted interventions and training for HCPs, including reception and administration staff, are crucial to ensure equitable, affirming cancer care for all LGBTQI patients, ultimately leading to improved satisfaction with cancer care.

**Implications for Cancer Survivors:**

Creating safe and inclusive environments for LGBTQI cancer patients is essential to encourage disclosure and improve satisfaction with cancer care.

**Supplementary Information:**

The online version contains supplementary material available at 10.1007/s11764-024-01677-1.

## Introduction

The experience of having cancer can pervade every area of a person’s life and cause considerable stress and distress [1]. For lesbian, gay, bisexual, transgender (trans), queer, and intersex (LGBTQI) people, the challenges of a cancer diagnosis, treatment, and survivorship are complicated by unique concerns and high rates of unmet needs [2–5], impacting satisfaction with cancer care [3, 6]. Satisfaction is an important indicator of quality of care, as satisfied patients report higher quality of life, treatment adherence, and continuity of care, leading to better health outcomes [7]. This is particularly relevant to LGBTQI people, who face intersectional, compounding disadvantages and minority stressors [8] resulting in unique risk factors for cancer and cancer-related distress compared with the non-LGBTQI population [3, 9]. For example, higher cancer burden among LGBTQI people is partly explained by higher rates of alcohol and smoking, which are a response to minority stress [10, 11], and lower rates of screening, associated with fear of discrimination in healthcare [9].

There is some evidence that sexual minority (i.e., lesbian, gay, bisexual, queer) cancer survivors have poorer satisfaction with care than their heterosexual peers [6, 12], with most research focused on those with breast [12] or prostate cancer [13, 14]. This low satisfaction with care reflects multiple systemic failings of cancer care systems to address the needs of LGBTQI communities [2] including the absence of LGBTQI inclusive policies and guidelines and the lack of LGBTQI visibility in clinical environments, on patient forms and in cancer service’s online presence [15]. Systemic failings are also evident in oncology healthcare professionals (HCPs) insufficient knowledge and confidence in treating LGBTQI patients [16, 17], lack of recognition and inclusion of LGBTQI partners and chosen family in clinical consultations [18], and the scarcity of tailored cancer information and support for LGBTQI populations [4, 19]. Moreover, pervasive cis-heterosexism and endosexism (negative beliefs and behaviors that privilege people who are cisgender, heterosexual and endosex (i.e., not intersex)) within the health system [4] result in lack of understanding and failure to address issues unique to LGBTQI communities [20, 21]. This includes the importance of sexuality orientation and gender identity (SOGI) disclosure [22], sexual concerns related to same-gender relationships [19, 23], impacts of minority stress [8], lack of support from biological family [24], distrust in healthcare systems arising from previous negative experiences [2, 25], and specific concerns of trans and intersex individuals [26, 27]. These elements contribute to feelings of invisibility and dissatisfaction among LGBTQI people undergoing cancer care [3, 28].

Disclosing SOGI or intersex variations in cancer care is a unique concern and decision for LGBTQI people and can be a major source of anxiety and dissatisfaction with care [12, 29]. While some LGBTQI patients do not consider their SOGI relevant to cancer care [12, 22], obviating the need for disclosure, for most people, non-disclosure creates an “additional burden of secrecy” [12, p. 1460] and leads to feelings of regret [30] that compound the already stressful experience of having cancer [31]. There are numerous benefits to LGBTQI disclosure, reported in general healthcare research, including increased satisfaction with care [32, 33], and greater healthcare engagement [34], participation in screening [35], mental health [36], and quality of life [37]. LGBTQI people who disclose in cancer care are also more likely to report authentic and holistic engagement with HCPs, leading to more tailored health information and support, and inclusion of their partners and chosen family [22]. However, these outcomes depend on a positive and accepting response to disclosure from HCPs [22].

Many LGBTQI patients fear discrimination [38] or inappropriate, ill-informed questions from HCPs, following disclosure of LGBTQI status [30, 39]. Concerns about sharing potentially stigmatizing personal information can be acute [40], with anticipated negative responses including aggressive reactions, religious talk, denial of the patient’s SOGI, or attempts to shame the discloser [41]. Some individuals undertake “preparatory work” [42, p. 1885] to reduce the likelihood of a negative response to disclosure, such as investigating the reputation of HCPs, seeking specific referrals, and avoiding care with religious providers. Preparatory work can also involve rehearsal of disclosure, incremental disclosure, and use of third-party disclosure [43], as well as preparing responses to the recipient’s reactions [44]. However, many LGBTQI people report difficulty challenging cis-heteronormative and endosexnormative assumptions in healthcare, inhibiting disclosure [4, 29]. The presence of a same-gender partner at appointments may facilitate disclosure for sexual minority people without the need for a direct statement [12, 22]. However, a lack of disclosure opportunities for patients without partners, and for trans and intersex people, can contribute to feelings of invisibility and lack of access to LGBTQI specific cancer information [22, 28]. Moreover, disclosure decision-making for trans and intersex participants may differ depending on what is salient to the medical interaction and what has been recorded in medical notes [45].

There are calls for HCPs to create opportunities for LGBTQI people to disclose in cancer care [20, 25, 46]. While there is a broad body of research examining LGBTQI disclosure in general healthcare [31, 32, 37, 47, 48] and more recently in the context of cancer care [4, 22, 29], the latter has predominately focused on sexual minority populations with breast [12, 42] or prostate cancer [30]. A knowledge gap persists regarding the optimal methods for cultivating safe and inclusive environments that encourage disclosure within cancer care settings, from the perspective of LGBTQI patients. More information is also needed to understand the decision-making and emotion work involved in disclosure for LGBTQI people in the context of cancer [40], as well as the association between disclosure and satisfaction with care, among LGBTQI people with various cancer types, age groups, and people who are trans (binary and non-binary) and have intersex variations [29]. To address these gaps in the literature, our research questions were as follows:

1. Is disclosure of LGBTQI status to HCPs and experiences of inclusivity and support from HCPs associated with satisfaction with cancer care across gender, sexuality, intersex status, and age groups?
2. How do LGBTQI people subjectively experience disclosure of sexuality identity, gender identity, and intersex status to cancer HCPS, including methods and reasons for disclosing or not disclosing their LGBTQI status?
3. What do LGBTQI patients want, in order to facilitate safe disclosure and improve satisfaction with care?

## Methods

### Study design and theoretical framework

This mixed-methods cross-sectional study was part of a broader program of research, The Out with Cancer Study, examining LGBTQI experiences of cancer and cancer care from the perspectives of LGBTQI people with cancer, caregivers, and HCPs [26, 28, 49–56]. This paper presents findings from an online survey and interviews with LGBTQI people with cancer about satisfaction with cancer care, including experiences of disclosure and what could be done to facilitate LGBTQI inclusive cancer care. The project was guided by an integrated knowledge translation (iKT) framework [57], facilitated through a stakeholder advisory group made up of 46 members, including LGBTQI people with cancer and caregivers, cancer HCPs, and representatives from LGBTQI health and cancer support organizations. This group was involved in co-design and co-production throughout all stages of the project. The study received ethics approval from the Western Sydney University Human Research Ethics Committee (ref. no. H12664), with secondary approval from ACON (formerly the AIDS Council of New South Wales) (ref. no. 2019/09).

### Participants and recruitment

Participants were eligible for this study if they (a) identified as LGBTQI, (b) had been diagnosed with cancer or had undergone a medical intervention related to cancer risk, and (c) were at least 15 years old. The inclusion of LGBTQI people who had undergone a medical intervention related to cancer risk was recommended by our stakeholder advisory group, as such individuals had interactions with cancer healthcare professionals. The inclusion of this group is particularly pertinent to intersex people who have innate variations of sex characteristics, many of whom have had medical interventions for a purported cancer risk [50].

The study was advertised across various platforms, including through the study partner organizations, other cancer and LGBTQI community organizations, cancer research participation databases such as Register 4 and ANZUP, cancer support networks, live LGBTQI gatherings, and social media platforms such as Facebook, X (formerly Twitter), and Instagram. Additionally, snowball sampling techniques were employed, encouraging participants to share the survey link with others who might be eligible for participation. Participant demographics were monitored, and recruitment strategies were refined through the data collection period to increase the recruitment of underrepresented groups. The study was open internationally, although recruitment focused on Australia and other English-speaking countries such as New Zealand, the UK, the USA, and Canada. Data were collected between September 2019 and September 2021.

### Measures

#### Quantitative measures

The survey was conducted online, facilitating anonymity, and included validated and unvalidated measures described elsewhere [56]. Separate survey pathways were asked in relation to sexuality identity, trans identity, and intersex variations, as relevant to participants. Relevant to the present study, satisfaction with care was examined using the following items.

Satisfaction with cancer care. Questions from ACON’s Sydney Women and Sexual Health Survey (SWASH) [58] and the Sydney Gay Community Periodic Survey [59] were used to assess participants’ satisfaction with care in relation to their being LGBTQI. Instead of the broad social groups included in the original scale, four items asked about satisfaction with oncology providers including medical HCPs, nursing HCPs, allied HCPs, and reception/administration staff. Participants responded using a five-point Likert scale (1 = *very dissatisfied –* 5 = *very satisfied*; *N/A*s *excluded*). Responses were averaged across survey pathways and HCP disciplines to provide an overall measure of satisfaction (range 1–5, higher scores indicating higher satisfaction).

##### LGBTQI disclosure to HCPs

The disclosure subscale of the Nebraska Outness Scale [60] was adapted to ask participants about the proportion of their cancer HCPs who were aware that they were LGBTQI. Mirroring the measure of satisfaction with care, four items were used to ask participants about their disclosure to oncology providers including medical HCPs, nursing HCPs, allied HCPs, and reception/administration staff. The original percentage response scale was replaced with a five-point Likert scale (*none/a few/some/ most/all/N/As excluded*) for ease of response and consistency with other survey items. As with the satisfaction measure, responses were averaged across survey pathways and HCP disciplines (range 1–5, higher scores indicating participants were more out to HCPs) to perform between-group comparisons and correlational analyses.

##### Means of disclosure

A single item asked participants how their HCPs became aware they were LGBTQI. Participants were provided with five possible options, which were derived from the previous literature on disclosure [13, 60].

##### Reasons for non-disclosure

An additional item asked about participants’ reasons for non-disclosure of LGBTQI status. Eleven possible options were provided, which were adapted from a previous study of gay and bisexual men with prostate cancer [13].

#### The consequences of patient disclosure were assessed through the following measures:

##### HCP acknowledgment of partner/s and support people

Two items were developed to assess the extent to which HCPs acknowledged participants’ partner/s and support people following disclosure. Participants responded using a five-point Likert scale (1 = *strongly disagree –* 5 = *strongly agree; N/As* excluded), with higher scores indicating greater HCP acknowledgment of partner/s and support people.

Discrimination in cancer care. A single item based on a previous study of sexual minority breast cancer survivors [8] was adapted to ask, “have you experienced discrimination as part of your cancer care.” Participants responded using a four-point Likert scale.

##### Ability to find information about cancer and being LGBTQI

A single item was developed to ask participants whether they could find helpful information about cancer and being LGBTQI. This item was originally derived from the Health Literacy Questionnaire [61], although given the limited availability of LGBTQI cancer information [52], this item was not interpreted as being indicative of participants’ health literacy.

#### Desire for information about cancer and being LGBTQI

Two items asked participants if they would like information about cancer and being LGBTQI and, if so, how they would like the information presented, adapted from a previous study of gay and bisexual men with prostate cancer [13].

#### Qualitative measures

At the end of each measure in the online survey, participants were asked the open-ended question, “*Is there anything you would like to tell us about this?*” Survey participants were also invited to take part in an interview to provide more insights into their experiences. The semi-structured interviews were conducted using an interview schedule and facilitated in a conversational style, each lasting 1 h. Researchers used video conferencing software, telephone or met in person, to conduct the interviews, and they were audio-recorded. Participants were asked about their experiences of cancer, including interactions with HCPs, decision-making about LGBTQI disclosure, and the consequences of this on their cancer care. They were also asked about the impact of cancer on their lives, including their identities and relationships, their experiences finding support networks and information, and their ideal cancer care.

### Analysis

In this mixed-methods study, quantitative data provided insight into the relationship between participants’ satisfaction with care and their experiences of disclosure, inclusivity and support from HCPs in cancer care. Qualitative data provided a rich, in-depth understanding of participants’ subjective experiences. The data were initially analyzed separately and then integrated [62] to provide a comprehensive understanding of the impact of disclosure in cancer care on LGBTQI patient satisfaction, facilitating analytic density [63].

#### Quantitative analysis

After the survey was closed, all responses were downloaded into IBM’s Statistical Package for the Social Sciences (SPSS) version 29 and screened to exclude surveys with insufficient, non-serious, or nonsensical responses; those from participants who were not LGBTQI; and repeated responses from the same participants (see Ussher et. al. [56], for details). Initial data inspection indicated that the study’s primary variables were within the acceptable ranges (skewness < 3, kurtosis < 10) [64].

To describe satisfaction with care (RQ1), disclosure, and experiences of inclusivity and support from HCPs, descriptive statistics (means, frequencies, percentages) were calculated for study variables. A series of analyses of variance (ANOVAs) and chi-square tests were used to compare these variables across LGBTQI groups (by gender, sexuality, and intersex status) and age groups (dichotomized as adolescent and young adult (AYA), aged 15–39 years, and older adult, 40 + years, following published recommendations for definition of AYA status [65]) and cancer types (dichotomized as reproductive and non-reproductive cancers). Comparison between reproductive and non-reproductive cancers was conducted to address the awareness that cancer of reproductive organs may necessitate disclosure of trans status or intersex variations. Furthermore, previous cancer research has largely examined breast and prostate cancers, and we wanted to explore if patterns around disclosure and satisfaction with care also apply to non-reproductive cancers. Finally, the association between participants’ satisfaction with care and experiences of cancer care variables (disclosure, HCP acknowledgment or partner/s and support people, ability to find LGBTQI cancer information, and discrimination in cancer care) were examined by exploring Pearson’s bivariate correlations between variables.

#### Qualitative analysis

The open-ended survey and interview data were analyzed using reflexive thematic analysis [66]. Within this framework, themes are developed through prolonged data immersion and reflection—an active and generative process [66]. Throughout the analysis, we engaged in a process of reflexivity, remaining aware our role in the knowledge production and reflecting critically on how our personal backgrounds, experiences and assumptions shaped the research process. Our research team and advisory group included individuals of diverse sexuality and gender identities, ages and cultural backgrounds. We also included both cancer survivors and those without personal cancer experiences encompassing a wide range of academic, professional, consumer, and community perspectives.

All interviews were transcribed and checked for accuracy. The transcripts were de-identified, and participant names were replaced with pseudonyms. The research team and several members of the stakeholder advisory group independently read line by line in close detail a subset of the open-ended surveys and interview data and made notes to identify first-order concepts or codes. Each team member brought suggestions of the first-order codes to a meeting and through a collaborative process of iterative discussion, first-order concepts with commonalities were grouped and organized into higher order codes, and a comprehensive coding framework was developed. This process allowed for defining and refining codes and consultation with the stakeholder advisory group on which data should be included within each code. Having formulated the coding framework, the data were imported into NVivo software to facilitate the organization of the qualitative data into relevant codes. Once coding was complete, each of the coded sections was summarized, which further helped to identify commonalities and facilitated theme development across the data set. Members of our stakeholder advisory group were again actively involved in reading and providing comments on the interpretation and reporting of the data. The themes were further revised to incorporate feedback on language and interpretation from the stakeholder advisory group. Throughout this process, the authors of this paper reflected upon how our identities as queer and non-queer, cis-gendered, White, middle-class, and Western researchers shaped the lens with which we bring to the analysis. In the presentation of results, LGBTQI people with cancer are identified by pseudonyms (for interviewees) or the word “survey,” with demographic details of age, sexuality, gender identity, intersex variations, and cancer type.

## Results

### Participant characteristics

A sample of 430 LGBTQI people who currently or had previously had cancer with a range of tumor types, and people who had undergone medical interventions for cancer risk, completed the survey. A subset of 104 participants, representing a cross-section of gender, sexuality, age, and tumor type, completed an interview. Table 1 presents the demographic and cancer characteristics of survey respondents. Most participants were cisgender, White, older adults, lived in Australia, and identified as lesbian, gay, or homosexual. There was greater diversity in terms of participants’ geographical location and types of cancer. A minority of participants were trans or non-binary, bisexual, queer, or reported an intersex variation. A minority identified as Australian Aboriginal, Torres Strait Islander or Māori (indigenous person of Aotearoa/New Zealand), and Asian or mixed ethnic background.

**Table 1:** Sociodemographic and cancer characteristics of participants

Demographic/professional characteristic	*M* (SD), range
Age at time of study (years) (*n* = 421)	52.6 (15.7), 16–92
Age at diagnosis (years) (*n* = 356)	46.3 (15.2), 1–79
Time since diagnosis (years)	7.1 (6.8), 0–43
	*n* (%)
Country (*n* = 430)	
*Australia*	311 (72.3%)
*United States of America*	62 (14.4%)
*United Kingdom*	29 (6.7%)
*New Zealand*	8 (1.9%)
*Canada*	7 (1.6%)
*Other country*	13 (3.0%)
Race/ethnicity (*n* = 425)	
*White*	362 (85.2%)
*Asian*	11 (2.6%)
*Australian Aboriginal, Torres Strait Islander or Măori*	9 (2.1%)
*Mixed background*	19 (4.5%)
*Other/unclear background*	24 (5.6%)
Location (*n* = 429)	
*Urban*	234 (54.5%)
*Regional*	145 (33.8%)
*Rural or remote*	50 (11.7%)
Gender (*n* = 430)	
*Cis female*	216 (50.2%)
*Cis male*	145 (33.7%)
*Trans (binary and non-binary)*^*a*^	63 (14.7%)
*Different identity*	6 (1.4%)
Sexuality (*n* = 430)	
*Lesbian, gay or homosexual*	317 (73.7%)
*Bisexual or pansexual*	47 (10.9%)
*Queer*	45 (10.5%)
*Straight or heterosexual*	10 (2.3%)
*Different or multiple identities*	11 (2.6%)
Intersex variation (*n* = 430)	
*Yes*	31 (7.2%)
*No*	388 (90.2%)
*Prefer not to answer*	11 (2.6%)
Cancer diagnosis (first) (*n* = 370)	
*Brain*	11 (3.0%)
*Breast*	90 (24.3%)
*Cervical*	11 (3.0%)
*Colorectal*	17 (4.6%)
*Head/neck*	14 (3.8%)
*Leukemia*	17 (4.6%)
*Lymphoma*	24 (6.5%)
*Ovarian*	17 (4.6%)
*Prostate*	59 (15.9%)
*Skin*	25 (6.8%)
*Uterine*	23 (6.2%)
*Other*	58 (15.7%)
*Not sure or unknown*	4 (1.1%)
Medical intervention for cancer risk	74 (17.2%)

^a^Non-binary (34, 7.9%), trans women (13, 3.0%), trans men (8, 1.9%), different trans identity (8, 1.9%)

### Association between satisfaction with cancer care and disclosure of LGBTQI status

#### Satisfaction with cancer care

When averaged across HCP disciplines, whilst participants were satisfied with their cancer care (*n* = 300, 76.3%), a notable 23.7% did report dissatisfaction. Satisfaction was significantly higher with nursing (*M* = 4.3, SD = 0.9), allied (*M* = 4.3, SD = 1.0), and medical (*M* = 4.3, SD = 1.0) professionals than for reception/administration staff (*M* = 4.2, SD = 1.0) (*F*_3,821_ = 4.125, *p* = 0.008). Significantly higher satisfaction was reported by cis men and women, compared to trans participants; by lesbian/gay participants, compared to bisexual and queer participants; by endosex participants without intersex variations, compared to those with intersex variations; and by older adults, compared to AYAs (Supplementary Table 1). There was no difference between reproductive and non-reproductive cancers.

#### LGBTQI disclosure to HCPs

The majority of participants had disclosed their LGBTQI sexuality or gender identity or intersex variations to some members of their cancer care team (*n* = 357, 87.1%). When averaged across disciplines, disclosure of patients’ sexuality identity (*M* = *3.5*, SD = 1.4) was more common than their trans identity (*M* = 3.2, SD = 1.6) or intersex variations (*M* = 2.8, SD = 1.3). Disclosure of patients’ LGBTQI status differed significantly by HCP discipline (*F*_*3*,906_ = 86.011, *p* < 0.001): allied health (*M* = 3.7, SD = 1.5) and medical (*M* = 3.7, SD = 1.5) professionals were more likely to have been made aware that patients were LGBTQI than nursing professionals (*M* = 3.5, SD = 1.5) and reception/admin staff (*M* = 2.8, SD = 1.6). Disclosure was highest among lesbian and gay participants compared to bisexual and queer participants, and among older participants compared to AYAs. Disclosure did not differ significantly by gender, intersex status, or cancer type (Supplementary Fig. 1).

#### Acknowledgment of partner/s and support people

The majority of participants agreed that their partner/s (*n* = 212, 76.8%) and support people (*n* = 257, 76.7%) were acknowledged by HCPs. Acknowledgment of partner/s was higher for lesbian/gay participants than for queer and bisexual participants and for those without intersex variations than for those with intersex variations, but did not differ significantly by gender or age. Acknowledgment of support people was higher for those without intersex variations than for those with intersex variations but did not differ significantly by gender, sexuality, or age (Supplementary Table 1).

#### Discrimination

A third of participants (*n* = 138, 33%) reported discrimination during their cancer care, because of being LGBTQI. This included 104 (31.0%) sexual minority participants, 22 (52.4%) trans participants, and 11 (50.0%) intersex participants. Trans participants reported significantly higher discrimination in cancer care compared to cisgender women and men. Additionally, significantly higher discrimination was reported by queer participants compared to gay/lesbian and bisexual participants; intersex participants in comparison to endosex participants; and by AYAs compared to older adults (Supplementary Table 1).

#### Ability to find information about *cancer* and being LGBTQI

Only 27.0% of sexual minority participants, 21.1% of trans participants, and 10.0% of participants with intersex variations reported being able to find helpful information about being LGBTQI and having cancer or medical intervention related to cancer risk. The ability to find helpful information about being LGBTQI and having cancer/medical intervention differed significantly by gender, sexuality, intersex status, and age (Supplementary Table 1).

#### Desire for information about cancer + being LGBTQI

Over half of sexual minority (59.5%) and trans (63.2%) participants and the majority of participants with intersex variations (85.0%) reported wanting information about their cancer (or cancer risk) and being LGBTQI. The desire for information was more common in AYAs (*n* = 58, 77.3%) than in older adults (*n* = 158, 56.6%; *χ*^2^ = 10.651, *p* = 0.001), but did not differ significantly by gender, sexuality, or intersex status.

#### Correlations between variables

Table 2 presents the correlations between satisfaction with care, disclosure, and experiences of inclusivity and support in cancer care. Satisfaction with care is significantly higher with more disclosure to HCPs, HCP acknowledgment of partner/s and support people, and the ability to find LGBTQI information about cancer. In contrast, satisfaction with care is lower with higher reports of discrimination in cancer care.

**Table 2:** Correlations between study variables

	Satisfaction with HCPs	Ability to find info	Discrimination in cancer care	Acknowledge partner/s	Acknowledge support people	Disclosure to HCPs
Disclosure to HCPs	.177***	.121*	.099	.258***	.205***	-
Acknowledgement of partner/s	.580***	.208***	− .344***	.718***	-	
Acknowledgement of support people	.450***	.211***	− .294***	-		
Discrimination in cancer care	− .518***	− .134**	-			
Ability to find LGBTQI cancer info	.246***	-				
Satisfaction with HCPs	-					

### Subjective experiences disclosing LGBTQI status to HCPs

#### Methods of disclosure and reasons for non-disclosure

Methods of disclosure and reasons for non-disclosure, based on closed-ended survey responses, are summarized in Table 3. The most common way in which HCPs learned about participants’ SOGI, or intersex variations was through self-disclosure during a consultation (sexual minority 67.5%; trans 54.8%; intersex 56.3%). For intersex participants, this was closely followed by their status being previously noted on their health records (43.8%). The most common reasons for not disclosing to HCPs for sexual minority participants were because it was not thought to be relevant or because discussions were focused on medical aspects of cancer. By contrast, trans participants were most likely to avoid disclosing their trans identity because of concern about discrimination, followed closely by them feeling embarrassed or uncomfortable. For participants with intersex variations, the most common reason for non-disclosure was concern about negative responses due to previous experiences with HCPs.

**Table 3:** Methods of disclosure and reasons for non-disclosure

	Sexual minority (*n* = 357)	Trans (*n* = 42)	Intersex (*n* = 16)
Methods of disclosure			
Self-disclosed during consultation	**67.5%**	**54.8%**	**56.3%**
Patient/medical forms gave option to say	17.0%	9.5%	6.3%
Health professional asked during consultation	5.3%	14.2%	0
Someone else told the healthcare professional	2.8%	7.1%	0
It was on the health records	0	14.2%	43.8%
Reasons for non-disclosure			
I felt embarrassed/uncomfortable to disclose	6.4%	26.1%	25.0%
I thought the healthcare professional would be embarrassed/uncomfortable	4.2%	9.5%	6.3%
It is a private matter	7.3%	9.5%	18.8%
I don’t think it is relevant	**37.8%**	23.8%	18.8%
I don’t think it would help	10.4%	11.9%	25.0%
I don’t know how to begin the discussion	6.4%	16.7%	18.8%
I was unsure that I was LGBTQI at the time	2.5%	7.1%	18.8%
I am worried about discrimination because of being LGBTQI	10.4%	**28.6%**	25.0%
I am worried about negative responses due to past experiences with healthcare professionals	7.8%	21.4%	**31.3%**
I don’t want my support person who attends appointments to know I am LGBTQI	4.2%	11.9%	0
Our discussions were entirely focused on the medical aspects of cancer	29.4%	23.8%	6.3%

In the thematic analysis of experiences of disclosure, two primary themes were identified: being out in cancer care and the importance of partner inclusion, each with sub-themes. The third theme relates to what patients want in relation to safe and inclusive cancer care.

#### Experiences of being “out” in cancer care

##### “This is me, accept it!” I am always “out”

Many participants attributed satisfaction with their cancer care to having their SOGI acknowledged by HCPs who “didn’t blink an eye” (Hector, 68, gay man, leukemia) and who accepted them “without any hesitation” (Eva, 61, lesbian woman, uterine cancer). Many participants, particularly older lesbian women, emphasized the importance of notifying their HCPs of their identity from the outset, “I’ve always been very out, so I always would disclose” (Bernice, 61, lesbian woman, breast cancer) and “it’s non-negotiable” (Beth, 59, lesbian woman, brain cancer). Ben, a 60-year-old gay man with oesophageal cancer, said, “It’s important for me to get it off my chest so they can understand me and how I operate. I basically do it so that they can give me appropriate care.” Participants said they felt able to “come out” in cancer care because “I’m a bit stroppy, like, I’m a bit in your face. If you don’t like it, then suck it up” (Patricia, 65, lesbian woman, uterine cancer) and “because we are older and have been out from the start of our journey this has assisted in being out with the medical profession” (Survey, 59, lesbian woman, brain cancer).

Participants used various strategies to “come out” to their cancer HCPs. Some said, “I don’t announce who I am. I just am who I am” (Alex, 35, non-binary, gay, testicular cancer). Others said they were automatically “outed” by the way they look, “I guess I look very gay, very, you know, outwardly. So, they would know straight away” (Jade, 34, lesbian woman, breast cancer). A number of trans participants reported that although they pass as cisgender, described as “stealthing” (Victor, 47, trans man, ovarian cancer), this was not possible when their cancer diagnosis did not align with anatomy typically associated with their gender. For instance, Victor (47, trans man, ovarian cancer) commented, “I wonder if I hadn’t had cancer if maybe I would have been a stealth trans man. But I’m not. I have ovarian cancer.” Conversely, some felt the need to explicitly verbalize their identity, as explained by Barbara, a 48-year-old lesbian woman with uterine cancer:

*I don’t necessarily always present as looking very traditionally queer. So I do have to verbalize that most of the time for people to realize or understand. I have no problem saying that to a doctor ever. I’ve always said it. But I don’t feel that I should have to say it more than once. I don’t feel that I should have to repeat that information, you know*.

A number of participants described being intentionally visible as LGBTQI patients in cancer care, recognizing the potential to positively influence attitudes despite the possibility of discrimination. Ash, a 40-year-old bisexual non-binary person with an unknown primary cancer, noted, “In the last year or so, I’ve actually been making a very deliberate choice to be more open and more visible” because “it adds up to shifting attitudes if I’m more visible.” Other participants spoke to the importance of being “open” and “challenging the system,” not only for themselves but “more for those others who might not have a voice to speak out” (Survey, 61, gay man, prostate cancer). These accounts illustrate the significance of LGBTQI disclosure for many patients in cancer care and some of the strategies utilized by participants to facilitate disclosure.

##### “That sweating moment”: the emotion work of disclosure

Participants﻿ of all ages, genders, sexualities, and intersex status, described difficulties deciding whether to share that they are LGBTQI with their cancer HCPs, as “you’ve got to decide whether to disclose or not disclose” (Paulette, 67, lesbian woman, colorectal cancer). Participants explained that “even though I am very experienced with it” (Marilyn, 76, lesbian woman, brain cancer) and “comfortable about my sexuality” (Survey, 50, lesbian woman, medical interventions for cancer risk), disclosing being LGBTQI, including correcting misassumptions, was “never easy” (Marilyn, 76, lesbian woman, brain cancer), could feel “awkward” (Cara, 29, gay woman, melanoma), “shaming” (Survey, 50, lesbian woman, medical intervention for cancer risk), “kind of quite scary” (Noel, 49, queer, non-binary, breast cancer), and required careful planning.

*I’ve always called coming out that sweating moment, you know, whether it’s to somebody that you’re friends with or want to be friends with, or it’s a provider. I do think carefully about the strategy. I want to always be in a good mood when I do it, I don’t want to be angry. I don’t want to feel particularly vulnerable. I want to be... sort of casually confident. Sometimes I pull it off better than others (Marilyn, 76, lesbian woman, brain cancer).*

While many participants reported that coming out to HCPs was easier when there were visible indicators of inclusivity, such as the “rainbow flag” or “trans flag,” some trans participants reported still feeling uncertain as “so many people put these stupid badges on because they think they’re friendly rather than because they actually know anything, and then get a shock [when our anatomy doesn’t align with our gender]” (Scott, 55, trans man, gay, multiple cancers).

Many participants, particularly younger people, feared negative reactions from HCPs, as Aaron (32, gay man, bowel cancer) questioned, “Will their potential negative reaction impact the quality of the care that I receive?” Participants explained that “constantly having to decide whether it’s worth disclosing to this person and whether that cost–benefit ratio of how much privacy you have to give up for your care is actually going to pay off” (Dylan, 32, gay, non-binary, leukemia) was exhausting. Paulette (67, lesbian woman, colorectal cancer) commented:

*So, you’re disclosing your sexuality the whole time, and you’re then leaving yourself open to people maybe not being particularly – I’m talking about medical professionals and stuff – not being too receptive, which can change their mindset and how they treat you. It just got a little bit wearing after a while, that continual coming out process.*

Some participants said their HCPs did not consider being LGBTQI relevant to cancer treatment and that mentioning such information unnecessarily complicated care, “sometimes I feel like there’s almost an unspoken pressure that you could streamline your care if you don’t bring that side to your consultations” (Dylan, 32, gay, non-binary, leukemia). Aaron (32, gay man, colorectal cancer) said, “It’s quite stressful when you sort of get that vibe from a specialist that it’s not really an area you can approach with them.” However, not sharing being LGBTQI with cancer HCPs led participants to “feel a bit awkward and uncomfortable. It’s not good. Not nice” (Cara, 29, gay woman, melanoma).

Participants said that receiving a positive response to their disclosure “was a relief and I thought “okay I can like you and open up” (Craig, 51, gay man, pancreatic cancer). However, others complained that even after coming out in cancer care, their disclosures weren’t acknowledged, “even after I had told various health professionals multiple times that I was a big old dyke they still kept assuming heterosexuality and asking about my ‘husband’” (Survey, 63, lesbian woman, breast cancer). Mary, a 54-year-old lesbian woman with breast cancer, said that coming out in cancer care “felt like it was back in the 80 s when I first came out, and health services absolutely didn’t do anything.” She explained that “now they’re not shocked when you tell them, but they still don’t do anything about it; it’s not automatically included in their work,” leading her to observe, “there’s no sense of inclusion, I guess.” These accounts highlight the persistent lack of awareness of SOGI identities in cancer care and the *work* required by LGBTQI patients to navigate disclosure.

##### “Forced into a glass closet”: reluctance to disclose

While some participants said they were not out in cancer care as “it never seemed like it mattered” (Brianna, 26, queer woman, lymphoma), others said, “I actually try to avoid it depending on who I’m talking to. I just sort of skirt around it, or I won’t even mention it” (Grace, 62, bisexual woman, cervical cancer) and “I had bad experiences with doctors around the time of my diagnosis so I try to avoid discussing it with medical professionals except where it’s clinically relevant” (Survey, 47, queer, non-binary, intersex, medical intervention for cancer risk). Carl, a 63-year-old gay man with prostate cancer, explained he was reluctant to disclose being LGBTQI during his cancer care because “I wasn’t out to anybody at that stage, not even my brother,” and Clark, a 60-year-old gay man with bladder cancer said, “I didn’t think it was relevant and I still have difficulty telling people.” Mary (54, lesbian woman, breast cancer) said that she wasn’t out with her cancer HCPs as she didn’t feel it was her “job” to take the lead in this type of discussion:

*Nobody said anything- and so neither did I. It’s not my job, that’s their job. I’m already in a heightened state of anxiety and, you know, not coping. It’s their job to put you at ease, not the other way around.*

Others disclosed being LGBTQI “on an as-needs basis” (Carter, 20, gay man leukemia), with some choosing to reveal only part of their identity depending on relevance. For instance, Ash (40, bisexual, non-binary, unknown primary cancer) explained, “I haven’t needed to disclose or mention that I’m poly because I haven’t dated that much around the peak periods of treatment time.”

Conversely, participants, particularly those in same-gender relationships reported feeling that they *had* to disclose that they are LGBTQI to their cancer HCPs, feeling it was necessary for effective care. Ellen, a 36-year-old lesbian woman with uterine cancer, explained:

*Getting cancer while lesbian is like forcing you into a glass closet. Prior to diagnosis, you know, our families knew, some close friends or select people at work knew. You know, we’re not actively hiding, but we’re not actively announcing either. It is kind of on a more need-to-know basis. I suppose. Whereas when you get cancer, I think you have to tell people, you have to tell your health professionals. Because that’s the first thing that you enter into. They have to know who the people are who are helping you and supporting you through it.*

Participants said that trying to conceal their same-gender relationship/s in cancer care could be difficult and acted as a barrier to their partners receiving support. As one survey participant said, “everywhere you go, you are asked who this person is with you, and eventually, you have to say so it gives it away. You can only lie so long” (57, asexual, trans man, medical interventions for cancer risk). He further explained that “saying we are just friends means they do not get access to many places or the information to help with decision-making.” These findings highlight the unique concerns and reluctance of some LGBTQI patients to disclose in cancer care and the difficulties of non-disclosure.

##### “There’s a lot of covert discrimination”: negative consequences of disclosure

Numerous participants said they felt that being out as LGBTQI impacted the cancer care they received, “there is a lot of covert discrimination. It’s a lot better than what it used to be, but it’s still there” (Glenn, 66, gay man, head/neck cancer) and “not everyone in the medical team was accepting or supportive” (Survey, 41, lesbian woman, uterine cancer). Patricia, a 65-year-old lesbian woman with uterine cancer, stated, “I think I’m quite assertive, I don’t hold back, and I’m very open about things. But I still think that being a lesbian impacted the care I received.” In these situations, some participants, particularly older LGBTQI people, negotiated changes to their cancer care teams, “If they had a problem, I found another provider” (Survey, 89, lesbian woman, breast cancer). A 64-year-old gay man with melanoma said:

*My first cancer skin specialist was fairly homophobic (very religious). I am also HIV*+*, and this added to his poor attitude. After he gave me a large dose of disdain and stigma I sacked him - went back to my very good GP - and requested a different referral. The GP practice I use no longer refers patients to this specialist.*

However, this wasn’t possible for all participants, as many needed to remain with their HCPs. Participants said that fear of prejudice and discrimination meant that clinical interactions with these HCPs were “just very transactional” (Aaron, 32, gay man, colorectal cancer) and left them feeling “in such a vulnerable position” (Barbara, 48, lesbian woman, uterine cancer). Intersex participants often reported “ignorance” (Survey, 57, queer man, intersex, medical intervention for cancer risk) from HCPs to their disclosure, with some participants explaining, “they (HCPs) are aware of my intersex variation, but don’t understand it at all” (Survey, 49, lesbian woman, intersex, medical intervention for cancer risk). These experiences highlight the many difficulties LGBTQI people with cancer may face in deciding if, when, and how to “come out” in cancer care, and the potential negative consequences of their disclosure.

#### Inclusion of LGBTQI partners and carers in *cancer* care

##### Same-gender partners help facilitate disclosure and need to be respected

In participants’ descriptions of their satisfaction with cancer care, the majority emphasized the importance of having their LGBTQI partners and carers acknowledged and involved in all aspects of care. The majority of sexuality and gender minority participants expressed the need for their “chosen family – family and community” (Paulette, 67, lesbian woman, colorectal cancer) to be “equally valued” (Riley, 53, lesbian woman, ovarian cancer) and to “feel comfortable, wanted and included” (Elaine, 68, lesbian woman, breast cancer) by HCPs. Participants said, “all the professionals I have seen have accepted my partner as my support person and person responsible” (Survey, 48, lesbian woman, melanoma) and “they included my partner in all the decision making and everything just as they I would assume with anybody else’s partner. She was quite accepted by the team” (Chloe, 65, lesbian woman, breast cancer).

For many participants, the presence of their LGBTQI partner/s at cancer appointments and during treatment helped to facilitate disclosure without the need for explicit verbalization. Martha, a 48-year-old lesbian woman with bowel cancer, said, “we’re not afraid to hold each other’s hand or walk into an office hand in hand at the Doctor or to be there for each other as any normal couple is,” and Rhonda, a 42-year-old lesbian woman with breast cancer said, “I just refer to my partner or just refer to the pronoun ‘her’ or something like that.” However, some people said they weren’t sure how to come out to their cancer HCPs when they didn’t have a same-gender partner. For instance, Craig, a 51-year-old gay man with pancreatic cancer, explained, “On the forms you get or the questions you get asked, “Do you have a partner?” No. “Do you have children?” No. And then it ends.” Leaving him to wonder, “Should I now, say I’m gay?”. Similarly, Lucy, a 75-year-old lesbian woman with uterine cancer, felt marginalized due to assumptions made based on her marital status, “I didn’t have a husband, I was divorced; therefore, that was all he [HCP] was interested in. I don’t think that he saw that there was any other option.”

##### “Is this your friend?” Lack of recognition of LGBTQI partners

Numerous participants reported being frustrated at HCPs who made incorrect assumptions about their relationship with their accompanying support person and “had a hard time understanding that this wasn’t just a friend” (Victor, 47, trans man, ovarian cancer). Cara, a 29-year-old gay woman with melanoma, said, “At my last MRI, the guy doing it, he sort of just assumed straight away that my partner was my friend, and it’s awkward to correct him. That’s happened on a few occasions.” Fynn, a 37-year-old queer non-binary person with uterine cancer, explained the pervasive assumption of cis-heterosexuality due to being married to a cis man, “people really usually automatically assume that I’m at least hetero.”

Participants expressed anger over HCP’s failure to recognize same-gender partners as legitimate carers, leading to their partner’s exclusion from important information and decision-making processes. A survey participant (42, lesbian woman, uterine cancer) said, “It was difficult for my partner to get any answers”; however, “when my parents turned up they were more than happy to talk to them. My family had to tell them that my partner is the one they should be speaking to.” To safeguard against this situation, Martha (48, lesbian woman, bowel cancer) said she would carry legal documentation with her “all of the time” to prove the nature of her relationship with her wife:

*Well, usually, when you first meet a new doctor or you go into surgery or whatever, you’ve always got to do your paperwork, which includes next of kin and all of that stuff. I always just say that she's my wife and enduring guardian, and we have those documents with us all the time.*

These accounts demonstrate the additional stressors and sources of distress that LGBTQI patients experience in cancer care.

#### “We want more than rainbow stickers”: achieving safe and inclusive cancer care

##### Diversity is part of HCP’s everyday thinking

Many participants expressed satisfaction with inclusive cancer care, describing their HCPs as “very accepting and very kind” (Fredrick, 57, gay man, prostate cancer), “absolutely brilliant” (Alicia, 65, lesbian woman, breast cancer), and “fantastic” (Archer, 59, lesbian woman, bowel cancer). Jake, a 30-year-old gay man with testicular cancer, said, “they were so awesome. The team that looked after me were top-notch.” When asked about what quality cancer care for LGBTQI people was, many participants spoke to the need for HCPs to be “supportive” (Max, 70, gay man, anal cancer), “non-judgemental” (Paulette, 67, lesbian woman, colorectal cancer), and have a “really high level of empathy” (Catherine, 61, bisexual woman, vulval cancer). Part of supportive and safe care was the request that HCPs do not make the “assumption” patients are heterosexual, cis-gender, or endosex (Christine, 53, lesbian woman, ovarian cancer), with acknowledgment of diversity being “mainstream” (Theresa, 55, lesbian woman, breast cancer) and part of “everyday thinking” (Survey, 55, lesbian woman, breast cancer). This included asking patients for their pronouns and mirroring terms they use for body parts, partners, and carers.

##### Fostering inclusive cancer care environments

Participants requested that HCPs engage in “sensitivity” (Johanna, 26, bisexual woman, lymphoma) and “awareness” (Ryan, 60, gay man, prostate cancer) training that goes beyond “whacking up a few rainbow stickers” (Survey, 55, pansexual, non-binary, intersex, skin cancer). Participants articulated the need for training that acknowledges differences between LGBTQI people (Ryan, 60, gay man, prostate cancer). Training must address the cancer care needs of trans and intersex people who are “long overdue” and “overlooked as a community” (Survey, 55, pansexual, non-binary, intersex, skin cancer). Participants suggested training could include “how to ask the right questions” (Paulette, 67, lesbian woman, colorectal cancer), recognition of unique “support structures” (Leonard, 58, gay man, prostate cancer) among LGBTQI communities, and enhance HCPs’ knowledge on how cancer treatment can affect gender and sexuality identity. Luca, a 33-year-old, queer, non-binary person with bladder cancer, highlighted the need for HCPs to consider their patient’s sexuality and gender identities “holistically.” They said:

*Understanding a person’s sexual identity preference and gender identity, like, I think you need to be looking at it holistically because you’re not just looking at the anatomy anymore. You’re looking at what that might mean to that person and what values and ideals they have behind it, which will change the treatment plan.*

Training is needed to move HCPs beyond the “theoretical,” as noted by Howard, a 33-year-old queer, trans man with uterine cancer who commented that “when it comes into practice, it’s so unusual to them that even though they’re trying to be respectful, it’s really new. They try to be respectful, but they have no fucking clue.” Undergraduate education at medical school was identified as an important avenue to begin training (Riley, 53, lesbian woman, ovarian cancer), but participants requested that training be “often” and “repeated” (Survey, 47, bisexual trans man, intersex, medical intervention for cancer risk).

Creating a non-discriminatory and visibly supportive healthcare environment was positioned as being important in increasing feelings of safety and inclusivity. Meaningful visual signifiers identified by participants included “the odd poster,” “gay pride flag,” and “rainbow stickers” (Glenn, 66, gay man, head/neck cancer). Participants also spoke of wanting “gender neutral signposting,” for example, not having all cancer posters “pink all the goddamn time” (Dylan, 32, gay, non-binary, leukemia). To enhance feelings of visibility, patients wanted resources, such as pamphlets in clinics, where they could see a “mirror image of you” (Dylan, 32, gay, non-binary, leukemia) and where patients could “see themselves reflected in the intake forms” (Victor, 47, straight, trans man, ovarian cancer). This included space for people to identify their name, “how you define yourself…your sexuality…your gender” (Leonard, 58, gay man, prostate cancer), whether you are “intersex” (Luca, 33, queer, non-binary, bladder cancer), ask “what is your partner’s name” (Glenn, 66, gay man, head/neck cancer) and that gives you the option to list “your pronoun” (Survey, 33, gay non-binary, leukemia).

##### LGBTQI inclusive information and support

Access to meaningful avenues for support and culturally safe information that addresses the needs of LGBTQI people and their carers was discussed as being critical, but was experienced as though “searching for a needle in the haystack” (Alex, 35, non-binary, gay, testicular cancer). In particular, many participants brought up the need for increased access to meaningful social support through LGBTQI-tailored support groups face-to-face, online, or through social media. This was positioned as being important because LGBTQI cancer patients and their carers have “slightly different focuses” (Kaye, 61, lesbian woman, ovarian cancer) that are often not recognized or addressed in mainstream cancer support groups. Patricia, a 65-year-old lesbian woman with uterine cancer, stated, “I just feel like my whole journey would have been so different if I had had an LGBTQI person to talk to about my concerns.” Similarly, participants desired more gender- and sexuality-specific cancer information across cancer survivorship topics, including sexuality and relationships and a wide range of cancer types. Participants particularly expressed the need for information about topics such as “safe sex during chemo,” noting that “everything is about heterosexual sex or relationships. Without this, patients described feeling “isolated” and “lost,” as exemplified in the following survey account “we have been so invisible and silent as a community and as we say, the “I” should be for Intersex inclusion, no more “I” for Invisible” (55, pansexual, non-binary, intersex, skin cancer). These findings demonstrate the pressing need for LGBTQI inclusive and tailored resources and support to address LGBTQI patients’ unmet needs and improve satisfaction with care.

## Discussion

The aim of this study was to examine the association between satisfaction with cancer care and patient disclosure of LGBTQI status, including the consequences of disclosure. We also explored subjective experiences of disclosure to HCPs, including the methods and reasons patients have for disclosing or not disclosing, and what LGBTQI cancer patients want in order to facilitate safe disclosure and improve overall satisfaction with care. This is the first large-scale study to specifically compare satisfaction with cancer care and LGBTQI disclosure within intersecting identity groups, including lesbian/gay, bisexual and queer, cisgender and trans, intersex and endosex, and AYAs and older adults, including a range of cancer types.

Our findings confirm previous reports in the general LGBTQI healthcare literature that disclosure to HCPs is related to higher satisfaction with care, compared to non-disclosure [33, 37, 67, 68]. Disclosure has been described as fundamental to achieving authenticity for LGBTQI people, but this is contingent on being responded to positively by HCPs [22]. Our study found that positive responses to disclosure, including HCP acknowledgment of partners and support people, access to tailored LGBTQI cancer information, and the absence of HCP discrimination, were significant factors in shaping patient satisfaction in cancer care. This reinforces the importance of establishing LGBTQI culturally safe practices as a central tenet of cancer care [20], including creating a safe and welcoming environment where disclosure is facilitated [69], to improve overall satisfaction and enhance health outcomes for LGBTQI cancer patients.

Self-disclosure during a consultation was the primary method of informing HCPs of LGBTQI status, confirming previous findings [37, 70], and suggesting that recommendations that HCPs facilitate disclosure by asking about SOGI and intersex variations on medical forms, or at first consultations [20, 46, 71], are not being followed for the majority of LGBTQI cancer patients. Decisions to disclose are an ongoing process over the lifespan, with daily decisions being made about when, to whom, and how to disclose [48, 72]. This is a social process described as “Personal Risking” [72], wherein a cost–benefit analysis is calculated of the risks associated with disclosure to HCPs, in order to manage disclosure-related fears. Cancer patients often have teams of health professionals involved in providing care and are burdened with the responsibility and emotion work of planning and initiating disclosure, anticipating HCP reactions, and rehearsing potential strategies [22, 25], as was confirmed by participants in the present study, where disclosure was described as a “sweating moment.” LGBTQI cancer patients report significantly higher distress than general cancer populations [56, 73]. Our results confirm that difficulties with disclosure contribute to the stress of cancer for many LGBTQI people and that positive, non-judgemental responses to disclosure from HCPs are essential for patient satisfaction.

In the absence of clear signals of inclusivity and proactive efforts by HCPs to create safe spaces and facilitate disclosure, non-disclosure can become the perceived safest option [42, 74], although is associated with poorer mental health and wellbeing in cancer care [75], and lower satisfaction with care. While the majority of participants had disclosed their SOGI or intersex variations to at least some of their cancer HCPs, non-disclosure was more common amongst trans, bisexual, queer, intersex, and AYA participants, and with reception/administration staff. This confirms previous reports of rates of non-disclosure being higher in bisexual women and men, compared to gay men and lesbian women [31], and provides vital information on disclosure in groups previously identified as neglected in LGBTQI cancer and disclosure research, trans, intersex, and young people [40, 76]. Reception/administration staff are often the first point of contact for patients in cancer care settings. They play a crucial role in communicating that the environment is safe and welcoming for LGBTQI patients and are instrumental in recording SOGI data [77]. Previous research has shown that sexuality- and gender-minority cancer patients who receive care in environments that are perceived as welcoming, and who feel safe disclosing their SOGI to their cancer care team, are six and three times, respectively, more likely to be satisfied with their care [43, 78]. Lower rates of satisfaction with care and higher rates of non-disclosure align with our prior findings of higher distress and lower quality of life among trans, bisexual, queer, intersex, and AYA LGBTQI people with cancer [56].

Reasons for non-disclosure varied by participant groups in our study. While over a third of sexual minority participants did not see the relevance of being LGBTQI to their cancer care, similar to previous research [12, 22], many still disclosed during the course of their treatment. This seeming contradiction may be explained by the “glass closet” metaphor used by one of our participants, an implicit pressure to disclose in order to achieve authenticity in cancer care interactions, access to information relevant to sexuality identities, and partner inclusion [22]. LGBTQI disclosure can help to build trusting relationships with HCPs, increasing the relevancy of information provided by both patients and HCPs and optimizing patient care [37]. The process of disclosure may be more difficult for younger people, who have less experience in disclosure in life, may be in a process of exploring their sexuality and gender identity, or may not be out to parents or other support people who attend consultations [49].

Non-disclosure among trans and intersex participants was more often due to fear of discrimination and concerns about negative responses, reflecting the unique social contexts and healthcare experiences of these groups [26, 27, 50], including higher rates of discrimination in healthcare [74, 79]. Our findings identify the very real concerns LGBTQI patients have about prejudice and discrimination from HCPs following disclosure, including the pathologization of identities, refusal of care, and receipt of substandard care [72]. These concerns are substantiated by accounts of HCP judgment, hostility, inappropriate questions, and exclusion of same-gender partners, with the highest rates of discrimination reported by trans and intersex participants. Past negative experiences of discrimination in healthcare [74] and broader societal prejudice [80], which are frequent experiences for LGBTQI individuals [8], also contribute to patients’ fears of negative reactions to disclosure from cancer HCPs.

Oncology HCPs report low confidence and limited knowledge about the needs of LGBTQI communities [16, 81], particularly for trans and intersex patients [51]. This manifests in a lack of HCP understanding of the psychosocial vulnerabilities of these communities [5, 73], low awareness of how treatment-related bodily changes may impact sexuality and gender identities [82], potential disruption of treatment on hormone therapies for intersex variations or gender affirmation [26], and cis-heteronormative assumptions that discourage disclosure and open communication, hindering optimal care [2–5].

LGBTQI patients were very clear about what they wanted from cancer care, through reflections on inclusive care and gaps in care. They wanted non-judgmental acceptance of SOGI and intersex variations and the absence of cis-heteronormative assumptions on the part of HCPs; inclusion of partners and other support people; training of HCPs in cultural safety when working with LGBTQI people; a safe environment where LGBTQI people were visible and included; and LGBTQI specific cancer information and support. These findings are in line with recommendations for LGBTQI culturally safe care outlined in systematic reviews of researcher and contained in training modules for HCPs in LGBTQI cancer care [69, 83–87]. Addressing gaps in LGBTQI cancer care can be achieved through co-designing LGBTQI cancer resources, securing dedicated funding, and tailoring existing cancer support groups to address LGBTQI people’s needs, and incorporating comprehensive training for healthcare professionals early and frequently in their education programs.

## Conclusions

In conclusion, our findings have significant implications for cancer HCPs and support services. Creating safe and inclusive environments for LGBTQI cancer patients is essential to encourage disclosure and improve satisfaction with cancer care [2, 28]. Therefore, HCPs, including reception/ administration staff, need to actively cultivate such environments by adopting practices that normalize LGBTQI inquiry [29]. These practices include asking about patients’ sexuality and gender identity and pronouns, providing inclusive options to record LGBTQI information on patient intake forms, providing positive, non-judgmental responses to LGBTQI disclosure, including partners and support people in care, and providing tailored LGBTQI cancer information [22, 25]. Additionally, targeted interventions are required, including curriculum development and professional training for oncology HCPs and that address the specific roles of reception/administration staff. These interventions should incorporate reflexive learning and concepts of cultural safety to improve HCP knowledge, confidence, and clinical practice behaviors [20]. Professional training is required alongside organisational system changes to create welcoming environments and LGBTQI inclusive institutional policies [20]. These actions are crucial to ensure HCPs avoid assumptions that patients are cisgender, heterosexual, and endosex and to better equip them to understand the unique needs of LGBTQI patients [69, 86, 87].

## Supplementary Information

Below is the link to the electronic supplementary material.

Supplementary file1 (DOCX 29 KB)

## Data Availability

The raw data supporting the conclusions of this article will be made available by the authors, without undue reservation.
