# Supplementary material for: “A sweating moment”: impact of disclosure in cancer care on LGBTQI patient satisfaction
Source: J Cancer Surviv. 2024 Sep 21;20(2):633–48. doi: 10.1007/s11764-024-01677-1 (PMC12988887; doi:10.1007/s11764-024-01677-1)
Supplement: Supplementary file 1 — Supplementary file1 (DOCX 29 KB) [file 11764_2024_1677_MOESM1_ESM.docx]

**Supplementary Information**

**Article Title:** “A sweating moment”: Impact of disclosure in cancer care on LGBTQI patient satisfaction

**Journal:** Journal of Cancer Survivorship

**Author names:** Rosalie Power, Jane Ussher, Kimberley Allison, Alexandra Hawkey, Janette Perz on behalf of The Out with Cancer Study Team

**Affiliation and email of corresponding author:** Rosalie Power (Translational Health Research Institute, Western Sydney University; r.power@westernsydney.edu.au)

# Supplementary Table 1: Differences by gender, sexuality, intersex status and age

|  | ***Gender*** | | | | |  | ***Sexuality*** | | | | | |  | | ***Intersex status*** | | | |  | ***Age*** | | | |
| --- | --- | --- | --- | --- | --- | --- | --- | --- | --- | --- | --- | --- | --- | --- | --- | --- | --- | --- | --- | --- | --- | --- | --- |
|  |  |  | *M (SD)* | | |  |  |  | *M (SD)* | | |  | |  | |  | *M (SD)* | |  |  |  | *M (SD)* | |
| **Outcome** | ***F*** | ***p*** | Cis female (n=216) | Cis male (n=145) | Trans  (n=63) |  | ***F*** | ***p*** | Lesbian/gay (n=317) | Bisexual (n=49) | Queer (n=47) |  | | ***F*** | | ***p*** | Intersex (n=30) | Endosex (n=381) |  | ***F*** | ***P*** | AYA  (n=90) | Older adult  (n=327) |
| Satisfaction with HCPs | 12.137 | **<.001** | 4.3 (0.8) | 4.4 (0.8) | 3.8 (1.0) |  | 14.863 | **<.001** | 4.4 (0.8) | 3.9 (0.8) | 3.8 (1.1) |  | | 61.235 | | **<.001** | 3.0 (1.1) | 4.4 (0.8) |  | 27.526 | **<.001** | 3.8 (1.0) | 4.4 (0.8) |
| Disclosure to HCPs | 1.840 | .160 | 3.4 (1.5) | 3.6 (1.3) | 3.3 (1.5) |  | 26.542 | **<.001** | 3.7 (1.3) | 2.2 (1.4) | 3.0 (1.5) |  | | 3.327 | | .069 | 2.9 (1.4) | 3.5 (1.4) |  | 33.907 | **<.001** | 2.6 (1.4) | 3.6 (1.4) |
| Acknowledgement of partner/s | 2.782 | .064 | 4.2 (1.1) | 4.2 (1.0) | 3.7 (1.1) |  | 10.919 | **<.001** | 4.3 (1.0) | 3.4 (1.2) | 3.8 (1.3) |  | | 5.664 | | **.018** | 3.6 (1.0) | 4.2 (1.0) |  | 11.504 | **.001** | 3.7 (1.2) | 4.2 (1.0) |
| Acknowledgment of support people | 2.594 | .076 | 4.1 (1.1) | 4.0 (1.0) | 3.7 (1.0) |  | 1.528 | .219 | 4.1 (1.1) | 3.8 (1.1) | 3.9 (1.1) |  | | 16.245 | | **<.001** | 3.2 (0.9) | 4.1 (1.0) |  | 0.079 | **.**779 | 4.0 (0.9) | 4.1 (1.1) |
| Discrimination in cancer care | 15.886 | **<.001** | 1.4 (0.6) | 1.4 (0.8) | 2.0 (1.1) |  | 6.556 | **.002** | 1.4 (0.8) | 1.3 (0.6) | 1.9 (1.1) |  | | 27.439 | | **<.001** | 2.2 (1.2) | 1.4 (0.7) |  | 7.413 | **.007** | 1.7 (0.9) | 1.4 (0.8) |
| Ability to find LGBTQI cancer info | 16.616 | **<.001** | 2.6 (1.1) | 3.2 (1.2) | 2.2 (1.3) |  | 3.396 | **.035** | 2.8 (1.2) | 2.4 (1.2) | 2.5 (1.4) |  | | 13.542 | | **<.001** | 1.9 (1.1) | 2.8 (1.2) |  | 12.082 | **.001** | 2.3 (1.2) | 2.8 (1.2) |

# Supplementary Figure 1. LGBTQI disclosure to HCPs
